# Onychoscopy in Nail Psoriasis: A Narrative Review of Diagnostic Utility, Disease Severity Assessment, Musculoskeletal Associations, and Treatment Monitoring

**DOI:** 10.7759/cureus.113912

**Published:** 2026-08-03

**Authors:** Karthika Sethumadhavan, Monika Sekar, K Srivenkateswaran

**Affiliations:** 1 Dermatology, Vinayaka Missions Medical College and Hospitals, Vinayaka Mission's Research Foundation (Deemed to be University), Puducherry, IND

**Keywords:** dermoscopy, nail psoriasis, onychoscopy, psoriatic arthritis, treatment monitoring

## Abstract

Nail psoriasis is a common and clinically significant manifestation of psoriasis that may be difficult to recognize, quantify, and differentiate from other nail disorders. Onychoscopy has emerged as a non-invasive imaging technique that enables detailed visualization of nail matrix, nail-bed, and vascular abnormalities. This narrative review critically synthesized the current evidence on the diagnostic and clinical applications of onychoscopy in nail psoriasis. Literature search of PubMed/MEDLINE, Scopus, and Google Scholar was conducted, and the available evidence indicates that pitting, onycholysis, subungual hyperkeratosis, splinter hemorrhages, leukonychia, and salmon patches are among the most consistently reported onychoscopic findings. Onychoscopy enhances the detection of subtle abnormalities compared with routine clinical examination and may complement severity assessment and differential diagnosis. Associations between selected nail abnormalities, psoriatic arthritis, and enthesitis suggest a potential role in identifying patients requiring closer musculoskeletal assessment, although predictive evidence remains insufficient. Evidence supporting serial onychoscopy for therapeutic monitoring is similarly limited. Considerable methodological heterogeneity, inconsistent terminology, and predominantly small cross-sectional studies restrict definitive conclusions. Standardized examination protocols, validated onychoscopic severity indices, and prospective multicenter studies are needed to establish the diagnostic, prognostic, and monitoring roles of onychoscopy in nail psoriasis.

## Introduction and background

Psoriasis is a chronic, immune-mediated inflammatory disease affecting approximately 2-3% of the global population and is increasingly recognized as a multisystem disorder involving the skin, nails, joints, and other organ systems [1]. Among its extracutaneous manifestations, nail psoriasis is one of the most frequent and clinically significant, occurring in nearly 50% of patients with psoriasis and affecting up to 80-90% during their lifetime, particularly among those with psoriatic arthritis (PsA) [2,3]. Nail involvement is associated with pain, impaired manual function, cosmetic disfigurement, psychological distress, and a substantial reduction in quality of life, emphasizing the need for early recognition and appropriate management [3,4].

The clinical manifestations of nail psoriasis depend on the anatomical site involved within the nail unit. Matrix involvement typically presents as pitting, leukonychia, and nail plate crumbling, whereas nail-bed disease is characterized by onycholysis, salmon patches, subungual hyperkeratosis, and splinter hemorrhages [3,4]. However, early or subtle nail changes may be difficult to detect on routine clinical examination, and differentiation from conditions such as onychomycosis, traumatic nail dystrophy, and nail lichen planus often remains challenging. Onycholysis represents a particular diagnostic challenge because it is a common manifestation of several nail disorders, including nail psoriasis, onychomycosis, traumatic nail dystrophy, and other inflammatory conditions. Considerable overlap in their clinical morphology may make accurate etiological diagnosis difficult on routine examination alone, potentially leading to unnecessary investigations, inappropriate treatment, delayed diagnosis, and treatment failure. In this context, onychoscopy may provide clinically useful, non-invasive morphological information that facilitates differentiation between common causes of onycholysis and guides the selection of patients requiring further mycological or histopathological evaluation.

Onychoscopy, the dermoscopic examination of the nail unit, has emerged as a simple, non-invasive imaging modality that enables detailed visualization of nail plate, matrix, nail-bed, hyponychial, and periungual structures beyond what is visible to the naked eye [5]. Increasing evidence suggests that onychoscopy improves diagnostic accuracy, facilitates objective assessment of disease severity using indices such as the Nail Psoriasis Severity Index (NAPSI), assists in differentiating nail psoriasis from its clinical mimics, and enables longitudinal monitoring of therapeutic response [5,6].

Although numerous observational studies have described individual onychoscopic findings in nail psoriasis, the available evidence remains heterogeneous and dispersed across its diagnostic and clinical applications, warranting a comprehensive synthesis of the current literature. Therefore, this narrative review aimed to critically synthesize the current evidence on the role of onychoscopy in the diagnosis, severity assessment, and clinical management of nail psoriasis. Specifically, the review sought to characterize the principal onychoscopic features of nail psoriasis, evaluate the added diagnostic value of onychoscopy over routine clinical examination and its role in differential diagnosis, examine the relationship between onychoscopic findings and established measures of nail and cutaneous disease severity, assess the associations between nail-unit abnormalities and PsA or related musculoskeletal involvement, evaluate the available evidence regarding the role of onychoscopy in monitoring therapeutic response, and identify current limitations, evidence gaps, and priorities for future research.

## Review

Methods

This narrative review was undertaken to provide a comprehensive and critical synthesis of the current evidence regarding the diagnostic and clinical applications of onychoscopy in nail psoriasis, including the characterization of onychoscopic features, disease severity assessment, differential diagnosis, associations with PsA and related musculoskeletal involvement, and therapeutic monitoring. The preparation and reporting of this review were informed by the principles of the Scale for the Assessment of Narrative Review Articles (SANRA), which promotes methodological transparency, balanced evidence synthesis, and scientific rigor in narrative reviews [7]. The literature search was designed to identify relevant evidence to support a structured narrative synthesis rather than to conduct an exhaustive systematic review. Accordingly, no protocol registration, duplicate independent screening, formal risk-of-bias assessment, or quantitative meta-analysis was undertaken.

A structured literature search was conducted between April 1 and June 30, 2026. PubMed/MEDLINE served as the primary bibliographic database, with articles searched from database inception to June 30, 2026. Supplementary searches were performed in Scopus to identify additional peer-reviewed publications and in Google Scholar to capture potentially relevant literature not retrieved through the primary database search. The reference lists of eligible publications and relevant review articles were also manually screened to identify additional pertinent literature.

Both Medical Subject Headings (MeSH) and free-text terms were used in various combinations with the Boolean operators "AND" and "OR." The principal search strategy combined terms related to nail psoriasis and onychoscopy and included ("Psoriasis" [MeSH] OR psoriasis OR "nail psoriasis") AND (onychoscopy OR dermoscopy OR "nail dermoscopy"). Minor modifications to the search syntax were made according to the indexing requirements and search functionality of the individual databases.

Publications were considered for inclusion if they addressed clinically relevant aspects of nail psoriasis, including dermoscopic or onychoscopic findings, disease severity assessment, early or isolated nail involvement, differential diagnosis, associations with PsA or related musculoskeletal manifestations, therapeutic monitoring, or other clinical applications of onychoscopy. Original observational studies, clinical trials, systematic reviews, meta-analyses, narrative reviews, evidence-based clinical guidelines, and expert consensus statements published in English and involving human participants were considered. Conference abstracts, editorials, correspondence, animal studies, non-English publications, and articles lacking sufficient methodological or clinical information to contribute meaningfully to the narrative synthesis were excluded. Case reports were generally excluded but were considered selectively when they described emerging or uncommon clinical applications for which higher-level evidence was unavailable.

Retrieved publications were reviewed for relevance using their titles, abstracts, and full texts where appropriate. Selection of the literature was guided primarily by relevance to the predefined clinical domains of this review and by the potential contribution of individual publications to the overall narrative synthesis rather than by formal systematic review procedures. The final narrative synthesis incorporated the most relevant evidence across the predefined clinical domains. When multiple publications addressed similar topics, greater emphasis was placed on systematic reviews, meta-analyses, clinical practice guidelines, consensus statements, and well-designed observational studies with clearly described methodology and adequate sample sizes. Rather than undertaking a formal risk-of-bias assessment, the methodological quality, clinical relevance, consistency of findings, and applicability of the available evidence were critically considered during the interpretative synthesis. This approach is consistent with the principles of SANRA and the methodology of high-quality narrative reviews [7,8].

Relevant information from the included publications was summarized, compared, and synthesized, including study design, patient characteristics, principal onychoscopic findings, methods of disease severity assessment, relationships with established severity indices such as the Psoriasis Area and Severity Index (PASI) and NAPSI, diagnostic and differential diagnostic applications, associations with PsA or related musculoskeletal involvement, therapeutic monitoring, and other clinically relevant findings. Owing to the methodological and clinical heterogeneity of the available literature, the evidence was synthesized narratively rather than quantitatively. Findings were critically compared across studies to identify recurring onychoscopic patterns, areas of agreement and disagreement, methodological limitations, and gaps in the existing evidence. The synthesis was organized into thematic sections encompassing the characteristics of the included studies, principal onychoscopic features, added diagnostic value, relationships with nail and cutaneous disease severity, associations with PsA and enthesitis, differential diagnosis, treatment monitoring, limitations of the available evidence, and priorities for future research.

Results

Characteristics of the Included Studies

The PubMed/MEDLINE search identified 210 records, and supplementary searches of Scopus and Google Scholar yielded 95 additional records. After removal of duplicates, 240 unique records were screened by title and abstract, of which 48 underwent full-text review; 16 publications met the eligibility criteria and were included in the final narrative synthesis, supplemented by three additional records identified through manual reference-list screening. The evidence base comprised predominantly cross-sectional and observational studies, supplemented by systematic and scoping reviews, a post hoc randomized trial analysis, and limited case-based evidence, with sample sizes ranging from 1 to 739 participants. The principal publications informing the major clinical domains of this review are summarized in Table 1, which includes 16 key studies and evidence syntheses. Most studies compared clinical and dermoscopic findings and assessed nail and cutaneous disease severity using the Nail Psoriasis Severity Index (NAPSI) and Psoriasis Area and Severity Index (PASI). Polat and Kapıcıoğlu [9], Asfiya et al. [10], Wanniang et al. [11], Varma et al. [12], Bindagi et al. [13], Long et al. [14], Arora et al. [15], Yorulmaz and Aksoy [16], Mashal et al. [17], Khokhar et al. [18], and Bhoi et al. [19] evaluated various aspects of onychoscopic morphology, diagnostic utility, disease severity, and associated musculoskeletal involvement. Considerable methodological heterogeneity was observed in patient selection, sample size, dermoscopic techniques, and outcome assessment. At the evidence-synthesis level, Rachadi and Chiheb [20] systematically reviewed 11 primary studies comprising 723 patients and summarized the principal dermoscopic manifestations of nail psoriasis.

**Table 1:** Characteristics and principal findings of key studies informing the clinical applications of onychoscopy in nail psoriasis DIP, distal interphalangeal; NAPSI, Nail Psoriasis Severity Index; PASI, Psoriasis Area and Severity Index; PsA, psoriatic arthritis; RCT, randomized controlled trial

Author, year	Study design	Evidence level	Population/sample	Clinical domain	Principal findings	Clinical relevance and key limitations
Polat and Kapıcıoğlu, 2017 [9]	Observational study	Cross-sectional observational	Patients with nail psoriasis (n=40)	Onychoscopic features and disease severity	Onychoscopy improved visualization of nail-bed and vascular abnormalities; clinical and dermoscopic NAPSI scores did not differ significantly.	Supports onychoscopy as a complementary assessment tool; incremental value for severity scoring was limited.
Asfiya et al., 2019 [10]	Observational study	Cross-sectional observational	Patients with nail psoriasis (n=50)	Onychoscopic findings and PsA	Dermoscopic findings differed between patients with and without arthritis, and onychoscopy provided additional information regarding nail involvement.	Suggests an association between nail abnormalities and articular involvement; predictive and prognostic significance remains uncertain.
Wanniang et al., 2020 [11]	Comparative observational study	Comparative observational	Patients with nail psoriasis (n=50)	Diagnostic utility and disease severity	Onychoscopy yielded higher NAPSI scores and improved visualization of salmon patches and splinter hemorrhages; PASI and NAPSI showed a weak positive correlation.	Supports enhanced detection of subtle nail abnormalities; the relationship between nail and cutaneous disease severity was weak.
Varma et al., 2021 [12]	Cross-sectional study	Cross-sectional observational	Patients with nail psoriasis (n=50)	Onychoscopic morphology	Multiple matrix, nail-bed, and vascular abnormalities, including characteristic capillary changes, were identified.	Contributes to morphological characterization of nail psoriasis; limited by the cross-sectional design and modest sample size.
Bindagi et al., 2021 [13]	Comparative observational study	Comparative observational	Patients with nail psoriasis (n=50)	Diagnostic utility and severity assessment	Onychoscopy improved detection of leukonychia, red spots in the lunula, onycholysis, and splinter hemorrhages and yielded higher onychoscopic NAPSI scores.	Supports the incremental value of onychoscopy for detecting and characterizing subtle nail abnormalities.
Long et al., 2021 [14]	Comparative observational study	Comparative observational	Patients with nail psoriasis and onychomycosis (n=135)	Morphology, disease severity, and differential diagnosis	NAPSI was positively associated with PASI and body surface area; selected dermoscopic abnormalities correlated with disease severity, and dermoscopic patterns assisted differentiation from onychomycosis.	Supports potential applications in severity assessment and differential diagnosis; diagnostic performance requires further validation against appropriate reference standards.
Arora et al., 2022 [15]	Observational study	Cross-sectional observational	Patients assessed using clinical and dermoscopic severity indices (n=100)	Severity assessment and joint involvement	NAPSI and dermoscopic NAPSI increased with PASI severity; modified clinical and dermoscopic NAPSI scores were associated with joint involvement.	Suggests potential utility of dermoscopic severity indices and possible identification of patients requiring closer musculoskeletal assessment.
Yorulmaz and Aksoy, 2022 [16]	Observational study	Cross-sectional observational	Patients with severe nail psoriasis (n=35)	Onychoscopic morphology and arthritis	Subungual hyperkeratosis and nail plate crumbling were more frequent among patients with arthritis; their combined presence was associated with an approximately 15-fold increased likelihood of arthritis.	Suggests a clinically relevant association between selected nail abnormalities and PsA; limited by the small sample and cross-sectional design.
Mashal et al., 2023 [17]	Observational study	Cross-sectional observational	Patients with psoriasis and nail involvement (n=50)	Onychoscopic morphology and disease severity	No significant correlation was observed between PASI and NAPSI; longitudinal striations and subungual hyperkeratosis were more frequent in moderate-to-severe psoriasis.	Highlights inconsistency in the relationship between nail and cutaneous disease severity.
Khokhar et al., 2023 [18]	Cross-sectional study	Cross-sectional observational	Patients undergoing clinical and onychoscopic assessment (n=50)	Added diagnostic value	Onychoscopy improved detection of pitting, leukonychia, onycholysis, and splinter hemorrhages and identified subtle abnormalities such as fuzzy lunula.	Supports the incremental value of onychoscopy for detecting abnormalities that may be inconspicuous on routine clinical examination.
Bhoi et al., 2023 [19]	Cross-sectional multimodal study	Cross-sectional observational	Patients undergoing clinical, onychoscopic, and ultrasonographic assessment (n=30)	PsA and enthesitis	DIP joint enthesitis was detected in 57% of digits with clinical nail involvement and was associated with nail thickening, crumbling, and onychorrhexis.	Suggests an association between nail abnormalities and subclinical musculoskeletal involvement; longitudinal and predictive significance remains uncertain.
Rachadi and Chiheb, 2024 [20]	Systematic review	Systematic review	11 studies comprising 723 patients	Onychoscopic morphology and diagnostic applications	Pitting and onycholysis were the predominant matrix and nail-bed findings, respectively; vascular abnormalities were reported in approximately 52% of patients.	Confirms recurring onychoscopic patterns while highlighting substantial methodological heterogeneity and the absence of standardized diagnostic criteria.
Rizzo et al., 2013 [21]	Descriptive study	Descriptive cross-sectional	Patients with psoriasis and nail involvement (n=43)	Differential diagnosis and coexisting onychomycosis	Positive fungal cultures were identified in 41.9% of patients with psoriatic nail involvement.	Highlights the potential coexistence of nail psoriasis and onychomycosis and supports complementary mycological investigation in diagnostically uncertain cases.
Megna et al., 2022 [22]	Case report with serial multimodal imaging	Case report	Pediatric patient with nail psoriasis (n=1)	Treatment monitoring	Clinical and dermoscopic abnormalities progressively improved during biologic therapy, with resolution accompanying clinical clearance.	Provides preliminary evidence for serial onychoscopic monitoring; generalizability is extremely limited.
Tillett et al., 2023 [23]	Post hoc analysis of an RCT	Post hoc RCT analysis	Patients from the VOYAGE 2 trial (n=739)	Treatment response	Biologic therapy produced substantial improvement in nail disease; early cutaneous response was associated with subsequent improvement in nail psoriasis.	Provides evidence regarding nail treatment response, although outcomes were primarily assessed using NAPSI rather than standardized serial onychoscopy.
Ortner et al., 2022 [24]	Systematic scoping review	Systematic scoping review	Non-invasive nail-imaging studies	Imaging techniques and terminology	Substantial heterogeneity in nail-imaging terminology was identified, with 244 reported feature descriptions condensed into 82 standardized terms.	Demonstrates substantial methodological and terminological heterogeneity and supports the need for standardized terminology and examination protocols.

Onychoscopic Features of Nail Psoriasis

The onychoscopic features of nail psoriasis reflected involvement of the nail matrix, nail bed, and surrounding vascular structures. Pitting was the predominant matrix finding, reported in 79.2-95% of patients in several studies, while other matrix abnormalities included leukonychia, nail plate thickening, transverse and longitudinal ridging, trachyonychia, crumbling, and lunular abnormalities [9,11-16,18]. Onycholysis was among the most frequent nail-bed findings, with a reported prevalence of up to 93.3%, while subungual hyperkeratosis, splinter hemorrhages, and salmon or oil-drop patches were also frequently identified [10-16,18]. Onychoscopy additionally demonstrated vascular abnormalities, including dilated and elongated nail-bed, nail-fold, and hyponychial capillaries [9,12,14]. The systematic review by Rachadi and Chiheb [20] similarly identified pitting as the predominant matrix abnormality and onycholysis as the most frequent nail-bed feature, with vascular abnormalities reported in approximately 52% of patients. Overall, although the prevalence of individual findings varied across studies, pitting, onycholysis, subungual hyperkeratosis, splinter hemorrhages, leukonychia, and salmon patches constituted the most consistently reported onychoscopic manifestations of nail psoriasis.

Added Diagnostic Value of Onychoscopy Over Clinical Examination

Onychoscopy consistently demonstrated greater sensitivity than routine clinical examination for detecting subtle nail abnormalities. Wanniang et al. [11] found significantly higher NAPSI scores on dermoscopic assessment, with salmon patches and splinter hemorrhages being better visualized than on clinical examination. Similarly, Bindagi et al. [13] reported improved detection of leukonychia, red spots in the lunula, onycholysis, and splinter hemorrhages, together with significantly higher onychoscopic NAPSI scores. Khokhar et al. [18] observed significantly greater detection of pitting, leukonychia, onycholysis, and splinter hemorrhages by onychoscopy and additionally identified subtle findings such as fuzzy lunula that were not apparent clinically. Although Polat and Kapıcıoğlu [9] found no significant difference between clinical and dermoscopic NAPSI scores, they reported improved visualization of nail-bed and vascular abnormalities. Collectively, these findings suggest that onychoscopy provides added diagnostic value by enhancing the recognition of subtle matrix, nail-bed, and vascular abnormalities that may be overlooked during routine clinical examination, particularly in patients with early, atypical, or isolated nail involvement.

Relationship With Nail and Cutaneous Disease Severity

The relationship between onychoscopic abnormalities and disease severity varied across studies. Long et al. [14] demonstrated a significant positive association between total NAPSI scores and both PASI and body surface area and identified several dermoscopic abnormalities, including red lunula, transverse grooves, nail plate crumbling, trachyonychia, oil-drop sign, erythematous borders of onycholytic areas, subungual hyperkeratosis, and dilated streaky capillaries, as correlates of nail disease severity. Arora et al. [15] similarly reported increasing NAPSI and dermoscopic NAPSI scores with increasing PASI severity and suggested that dermoscopic severity indices may improve the assessment of nail involvement. Bindagi et al. [13] and Wanniang et al. [11] also observed positive relationships between cutaneous and nail disease severity, although the latter reported only a weak correlation between PASI and NAPSI scores. Conversely, Polat and Kapıcıoğlu [9] and Mashal et al. [17] found no significant correlation between PASI and NAPSI scores, although Mashal et al. identified longitudinal striations and subungual hyperkeratosis more frequently in moderate-to-severe psoriasis. Thus, while several studies suggest that greater nail involvement and specific onychoscopic abnormalities may reflect increasing psoriasis severity, the relationship between nail and cutaneous disease severity remains inconsistent across the available literature.

Association With Psoriatic Arthritis and Enthesitis

Nail involvement and specific onychoscopic abnormalities may provide clinically relevant information regarding associated PsA and enthesitis. Asfiya et al. [10] observed differences in dermoscopic findings between patients with and without arthritis, suggesting that onychoscopy combined with NAPSI assessment may contribute to the evaluation of articular involvement. Arora et al. [15] similarly reported that modified clinical and dermoscopic NAPSI scores were significant predictors of joint involvement. In patients with severe nail psoriasis, Yorulmaz and Aksoy [16] found that subungual hyperkeratosis and nail plate crumbling occurred more frequently among patients with arthritis, with their combined presence associated with a 15-fold increase in arthritis risk. Bhoi et al. [19] further demonstrated ultrasonographic evidence of distal interphalangeal joint enthesitis in 57% of digits with clinical nail involvement, including clinically asymptomatic patients, and found significant associations between enthesitis and nail thickening, crumbling, and onychorrhexis. Collectively, these findings suggest a relationship between nail-unit abnormalities and underlying musculoskeletal involvement, although the available evidence is predominantly derived from small cross-sectional studies and does not establish the predictive value of onychoscopy for future PsA development.

Differential Diagnosis

Onychoscopy may facilitate the differentiation of nail psoriasis from clinically similar nail disorders, particularly onychomycosis, although overlap between conditions remains an important diagnostic consideration. Long et al. [14], in a comparative study of patients with nail psoriasis and onychomycosis, demonstrated that detailed assessment of dermoscopic patterns could improve diagnostic accuracy for nail psoriasis. The systematic review by Rachadi and Chiheb [20] similarly identified recurring onychoscopic patterns, including pitting, onycholysis, oil-drop patches, erythematous borders of onycholytic areas, splinter hemorrhages, subungual hyperkeratosis, and vascular abnormalities, which may collectively support the diagnosis of nail psoriasis. However, Rizzo et al. [21] demonstrated positive fungal cultures in 41.9% of patients with psoriatic nail involvement, highlighting the potential coexistence of onychomycosis and nail psoriasis and the limitations of relying exclusively on clinical morphology. Overall, onychoscopy provides a useful non-invasive adjunct for identifying patterns suggestive of nail psoriasis and distinguishing it from common mimics, but complementary mycological or histopathological investigations may remain necessary in diagnostically uncertain cases.

Role of Onychoscopy in Treatment Monitoring

Evidence regarding the role of onychoscopy in monitoring therapeutic response in nail psoriasis remains limited compared with its diagnostic and severity-assessment applications. Most treatment studies have evaluated therapeutic response using changes in clinical NAPSI rather than serial onychoscopic findings. Megna et al. [22] reported progressive clinical, dermoscopic, and reflectance confocal microscopic improvement in nail psoriasis during biologic treatment, with resolution of dermoscopic abnormalities accompanying complete clinical clearance. However, the evidence was derived from a single pediatric case and therefore has limited generalizability. Tillett et al. [23], in a post hoc analysis of the VOYAGE 2 randomized trial, demonstrated substantial improvement in nail disease during biologic therapy and found that early improvement in cutaneous psoriasis was associated with subsequent nail response, although therapeutic outcomes were primarily assessed using NAPSI rather than onychoscopy. Overall, direct evidence supporting serial onychoscopy for treatment monitoring remains scarce, and prospective longitudinal studies using standardized onychoscopic outcomes are required to determine its clinical value in assessing therapeutic response.

Discussion

The findings of this narrative review support onychoscopy as a useful non-invasive adjunct in the evaluation of nail psoriasis, particularly for enhanced morphological characterization and detection of abnormalities that may be inconspicuous on routine clinical examination [9,11,13,19]. Available evidence also suggests potential applications in severity assessment, differential diagnosis, identification of nail changes associated with musculoskeletal involvement, and therapeutic monitoring [13-16,19,22,23]. However, the strength of evidence across these applications remains unequal. While the morphological and diagnostic utility of onychoscopy is supported by relatively consistent observational evidence, its roles in standardized severity assessment, prediction of PsA, and therapeutic monitoring remain insufficiently validated.

The spectrum of onychoscopic abnormalities reflects involvement of different anatomical components of the nail unit. Pitting and onycholysis are the predominant manifestations of nail matrix and nail-bed involvement, respectively [11-14,17,20], while other matrix, nail-bed, and vascular abnormalities further illustrate the heterogeneous manifestations of psoriatic nail disease [9-16,18]. The reported prevalence of individual findings varies considerably, probably reflecting differences in patient characteristics, disease severity, examination techniques, and definitions of onychoscopic abnormalities. Although the systematic review by Rachadi and Chiheb [20] confirmed several recurring morphological patterns, substantial heterogeneity across studies underscores the need for standardized terminology and examination protocols.

The principal clinical advantage of onychoscopy is its ability to reveal subtle abnormalities that may be overlooked during routine examination. Improved visualization of leukonychia, splinter hemorrhages, salmon patches, onycholysis, and lunular abnormalities, together with higher dermoscopically assessed NAPSI scores in some studies, suggests that conventional examination may underestimate the extent of nail involvement [11,13,18]. This incremental value may be particularly relevant in patients with early, atypical, or isolated nail disease.

Nevertheless, improved detection should not be equated with definitive diagnostic accuracy. Most available studies compared morphological findings identified by clinical and onychoscopic examination rather than evaluating formal diagnostic performance against an independent reference standard. Furthermore, individual onychoscopic abnormalities are not specific to psoriasis and may overlap with fungal, inflammatory, and traumatic nail disorders [14,20,21]. Onychoscopy should therefore be regarded as a complementary extension of clinical examination rather than a replacement for mycological testing or histopathological assessment when diagnostic uncertainty persists.

The relationship between onychoscopic abnormalities and established measures of nail and cutaneous disease severity remains inconsistent. Several studies reported positive associations between NAPSI and PASI and between selected dermoscopic findings and greater disease severity [10,13-15], whereas others demonstrated weak or nonsignificant correlations [9,11]. These discrepancies may reflect differences in study populations, sample sizes, disease severity, examination techniques, and severity indices. Nail and cutaneous manifestations may also represent related but partially independent dimensions of psoriatic disease activity, which could explain why their severity does not consistently parallel each other. Although onychoscopy may complement conventional severity assessment by detecting subtle abnormalities, validated dermoscopic severity indices demonstrating reliability, responsiveness to treatment, and association with clinically meaningful outcomes are required before routine clinical implementation.

The relationship between nail abnormalities and musculoskeletal involvement represents an important emerging application of onychoscopy. The close anatomical relationship between the nail unit, distal interphalangeal joint, and extensor tendon enthesis provides biological plausibility for the association between nail psoriasis and PsA. Associations between selected nail abnormalities, arthritis, and ultrasonographically detected enthesitis further support this relationship [10,15,16]. Subungual hyperkeratosis, nail plate crumbling, nail thickening, and onychorrhexis have been associated with articular or entheseal involvement [16,19], while the detection of distal interphalangeal joint enthesitis in clinically asymptomatic patients suggests that nail abnormalities may coexist with subclinical musculoskeletal inflammation [18]. However, the predominantly cross-sectional design and small sample sizes of available studies preclude conclusions regarding the ability of these findings to predict future PsA. Selected nail abnormalities may therefore justify closer assessment for musculoskeletal symptoms and rheumatological evaluation when clinically appropriate, but their use as predictive biomarkers remains premature.

Onychoscopy may also assist in differentiating nail psoriasis from clinically similar disorders, particularly onychomycosis. Combinations of matrix, nail-bed, and vascular abnormalities are likely to provide greater diagnostic value than reliance on individual findings [14,20]. However, considerable morphological overlap exists, and no single onychoscopic feature should be regarded as independently diagnostic. The coexistence of psoriasis and fungal nail infection further complicates diagnostic assessment. Rizzo et al. [21] reported positive fungal cultures in 41.9% of patients with psoriatic nail involvement, demonstrating that dystrophic nail changes in patients with psoriasis cannot automatically be attributed to psoriatic disease. Onychoscopy is therefore best used to refine clinical assessment and guide further investigation, with mycological examination remaining important when fungal infection is suspected and histopathological assessment reserved for selected diagnostically uncertain cases.

Evidence supporting onychoscopy for therapeutic monitoring remains preliminary because most treatment studies assess nail response using clinical NAPSI rather than standardized serial dermoscopic evaluation [22,23]. Nevertheless, onychoscopy may provide an objective means of documenting morphological improvement and identifying persistent abnormalities during treatment. Prospective longitudinal studies using standardized imaging protocols and predefined dermoscopic outcomes are required to establish whether serial onychoscopy provides clinically meaningful information beyond conventional severity assessment.

Strengths and Limitations

This review provides a broad clinical synthesis encompassing morphological characterization, diagnostic utility, severity assessment, associations with PsA and enthesitis, differential diagnosis, and therapeutic monitoring. Additional strengths include a structured literature search, application of SANRA principles, and critical synthesis of heterogeneous evidence. Several limitations should be acknowledged. The narrative methodology and the absence of formal risk-of-bias assessment may have introduced selection and interpretative bias, while restriction to English-language publications and potential publication bias may have limited the comprehensiveness of the evidence base. The inclusion of heterogeneous publication types, ranging from primary observational studies to evidence syntheses and limited case-based evidence, may also have contributed to variability in the strength and interpretation of the available evidence. Furthermore, substantial heterogeneity in patient selection, sample size, dermoscopic techniques, terminology, and outcome assessment restricted direct comparison and precluded quantitative pooling. The predominance of small, single-center, cross-sectional studies particularly limits conclusions regarding the prognostic and longitudinal applications of onychoscopy.

Future Research Directions

Future research should prioritize standardized onychoscopic terminology, image-acquisition protocols, and validated dermoscopic severity indices, as the variability in techniques and nomenclature reported across the literature limits reproducibility [24]. Multicenter prospective studies are needed to establish the incremental diagnostic value of onychoscopy over conventional examination and to determine interobserver and intraobserver reliability against appropriate reference standards. Longitudinal studies should further assess whether specific onychoscopic abnormalities predict incident PsA and whether standardized serial onychoscopy adds clinically meaningful information for treatment monitoring beyond established severity indices, with integration of automated image-analysis approaches offering a potential route toward greater objectivity [24].

## Conclusions

Current evidence supports onychoscopy as a useful adjunct to clinical examination in nail psoriasis, particularly for detecting and characterizing subtle matrix, nail-bed, and vascular abnormalities that may be underestimated on routine examination. Its most established role lies in enhanced morphological assessment, whereas evidence for its use in standardized severity evaluation, differentiation from clinical mimics, identification of patients with underlying musculoskeletal involvement, and therapeutic monitoring remains less robust. Importantly, current studies do not establish onychoscopic findings as diagnostic criteria or predictors of future PsA, and onychoscopy should not replace mycological or histopathological investigation when diagnostic uncertainty persists. The predominance of small, cross-sectional studies, together with heterogeneous terminology, imaging techniques, and outcome measures, limits the clinical generalizability of the available evidence. Future multicenter prospective studies using standardized imaging protocols and validated assessment tools should determine the incremental diagnostic value, reliability, prognostic significance, and responsiveness to treatment of onychoscopic findings. Until such evidence is available, onychoscopy is best positioned as a complementary clinical tool that enhances the assessment of nail psoriasis while its broader diagnostic, prognostic, and monitoring applications remain to be established.

## References

[1] Parisi R, Iskandar IY, Kontopantelis E, Augustin M, Griffiths CE, Ashcroft DM (2020). National, regional, and worldwide epidemiology of psoriasis: systematic analysis and modelling study. BMJ.

[2] Kaeley GS, Eder L, Aydin SZ, Rich P, Bakewell CJ (2021). Nail psoriasis: diagnosis, assessment, treatment options, and unmet clinical needs. J Rheumatol.

[3] Klaassen KM, van de Kerkhof PC, Pasch MC (2013). Nail psoriasis: a questionnaire-based survey. Br J Dermatol.

[4] Stewart CR, Algu L, Kamran R, Leveille CF, Abid K, Rae C, Lipner SR (2021). The impact of nail psoriasis and treatment on quality of life: a systematic review. Skin Appendage Disord.

[5] Hirata SH, Yamada S, Almeida FA, Enokihara MY, Rosa IP, Enokihara MM, Michalany NS (2006). Dermoscopic examination of the nail bed and matrix. Int J Dermatol.

[6] Alessandrini A, Starace M, Piraccini BM (2017). Dermoscopy in the evaluation of nail disorders. Skin Appendage Disord.

[7] Baethge C, Goldbeck-Wood S, Mertens S (2019). SANRA-a scale for the quality assessment of narrative review articles. Res Integr Peer Rev.

[8] Gasparyan AY, Ayvazyan L, Blackmore H, Kitas GD (2011). Writing a narrative biomedical review: considerations for authors, peer reviewers, and editors. Rheumatol Int.

[9] Polat A, Kapıcıoğlu Y (2017). Dermoscopic findings of psoriatic nail and their relationship with disease severity. Turkderm.

[10] Asfiya A, Shanavaz AA, Shenoy M, Vishal B (2019). Utility of dermoscopy in nail psoriasis: its correlation with the Nail Psoriasis Severity Index (NAPSI) and psoriatic arthritis. Indian J Clin Exp Dermatol.

[11] Wanniang N, Navya A, Pai V, Ghodge R (2020). Comparative study of clinical and dermoscopic features in nail psoriasis. Indian Dermatol Online J.

[12] Varma K, Kumar U, Jain P (2021). Dermoscopic evaluation of nail psoriasis: a cross-sectional study. Int J Res Dermatol.

[13] Bindagi AP, Doshi B, Pandit A, Manjunathswamy B (2021). Nail changes in psoriasis: correlation between onychoscopy and NAPSI scoring. J Psoriasis Psoriatic Arthritis.

[14] Long D, Zhang Z, He F (2021). Dermoscopic features of nail psoriasis: positive correlation with the severity of psoriasis. J Dermatol.

[15] Arora S, Paul D, Kumar R, Bhatnagar A, Arora G, Mech S, Suhag DK (2022). Study of nail psoriasis and dermoscopic correlation with dermoscopic and modified dermoscopic nail psoriasis severity indexes (dNAPSI and dmNAPSI). Dermatol Pract Concept.

[16] Yorulmaz A, Aksoy GG (2022). Dermoscopic features of nail psoriasis: revisited. Skin Appendage Disord.

[17] Mashal ZR, Elgamal EE, Zaky MS, Elsaie ML (2023). Dermoscopic features of psoriatic nails and their correlation to disease severity. Dermatol Res Pract.

[18] Khokhar R, Mehta RD, Ghiya BC (2023). Clinical and onychoscopic evaluation of nail changes in psoriasis at a tertiary-care hospital: a cross-sectional study. Our Dermatol Online.

[19] Bhoi AK, Grover C, Singal A, Tandon A (2023). Enthesopathy in patients with nail psoriasis - a cross-sectional evaluation of clinical, onychoscopic and ultrasonographic features. Indian J Dermatol Venereol Leprol.

[20] Rachadi H, Chiheb S (2024). Dermoscopic features of nail psoriasis: a systematic review. Int J Dermatol.

[21] Rizzo D, Alaimo R, Tilotta G, Dinotta F, Bongiorno MR (2013). Incidence of onychomycosis among psoriatic patients with nail involvement: a descriptive study. Mycoses.

[22] Megna M, Villani A, Potestio L, Camela E, Fabbrocini G, Ocampo-Garza SS (2022). Adalimumab biosimilar in a pediatric patient: clinical and in vivo reflectance confocal microscopy evaluation. Dermatol Ther.

[23] Tillett W, Egeberg A, Sonkoly E (2023). Nail psoriasis dynamics during biologic treatment and withdrawal in patients with psoriasis who may be at high risk of developing psoriatic arthritis: a post hoc analysis of the VOYAGE 2 randomized trial. Arthritis Res Ther.

[24] Ortner VK, Mandel VD, Bertugno S, Philipsen PA, Haedersdal M (2022). Imaging of the nail unit in psoriatic patients:A systematic scoping review of techniques and terminology. Exp Dermatol.

